# Climate warming enhancement of catastrophic southern California debris flows

**DOI:** 10.1038/s41598-020-67511-7

**Published:** 2020-06-29

**Authors:** Diandong Ren, Lance M. Leslie

**Affiliations:** 1 School of Mathematical and Physical Sciences, University of Technology Sydney, PO Box 123, Broadway, Sydney, 2007 New South Wales Australia; 2 School of Electrical Engineering, Computing and Mathematical Sciences, Curtin University, Kent St, Perth, 6845 WA Australia; 3 School of Mathematical and Physical Sciences, UTS, Sydney, Australia

**Keywords:** Hydrology, Environmental impact

## Abstract

The sequence of wildfires followed by debris flows, frequently affects southern California, reflecting its drought-heavy precipitation climate bipolarity. Organic debris from incomplete burning is lighter than inorganic matter, and partially inviscid. Hence lower rainfall totals can trigger downslope motion than typically required by the underlying clasts of loose inorganic granular material. After advection downslope, the pebble-laden organic debris has a higher capacity for rilling; a positive feedback process. A mechanism is proposed whereby boulders are ‘rafted’ by organic debris. This coordinated movement of boulders greatly enhances the debris flow erosion capacity. This climate change sensitive debris flow enhancing mechanism, through organic–inorganic granular material interaction, is supported by observations and the numerical simulations. Using a model explicitly parameterizing erosion processes, including runoff entrainment, rilling incision, and bank collapse, the lifecycle of the Montecito debris flow of January 9, 2018 is simulated, providing quantitative estimates of mass conveyed and debris sorting at the terminus. Peak rafting speeds are ~ 12.9 m/s at ~ 300 m asl. Total boulder (effective diameter > 25 cm) volume involved for the Ysidro Creek area alone is ~ 5 × 10^4^ m^3^, scattered between the region 50–260 m asl. Debris flows are highly repeatable and locations prone to debris flows are identified and their likelihood of realization estimated.

## Introduction

**Figure 1:**
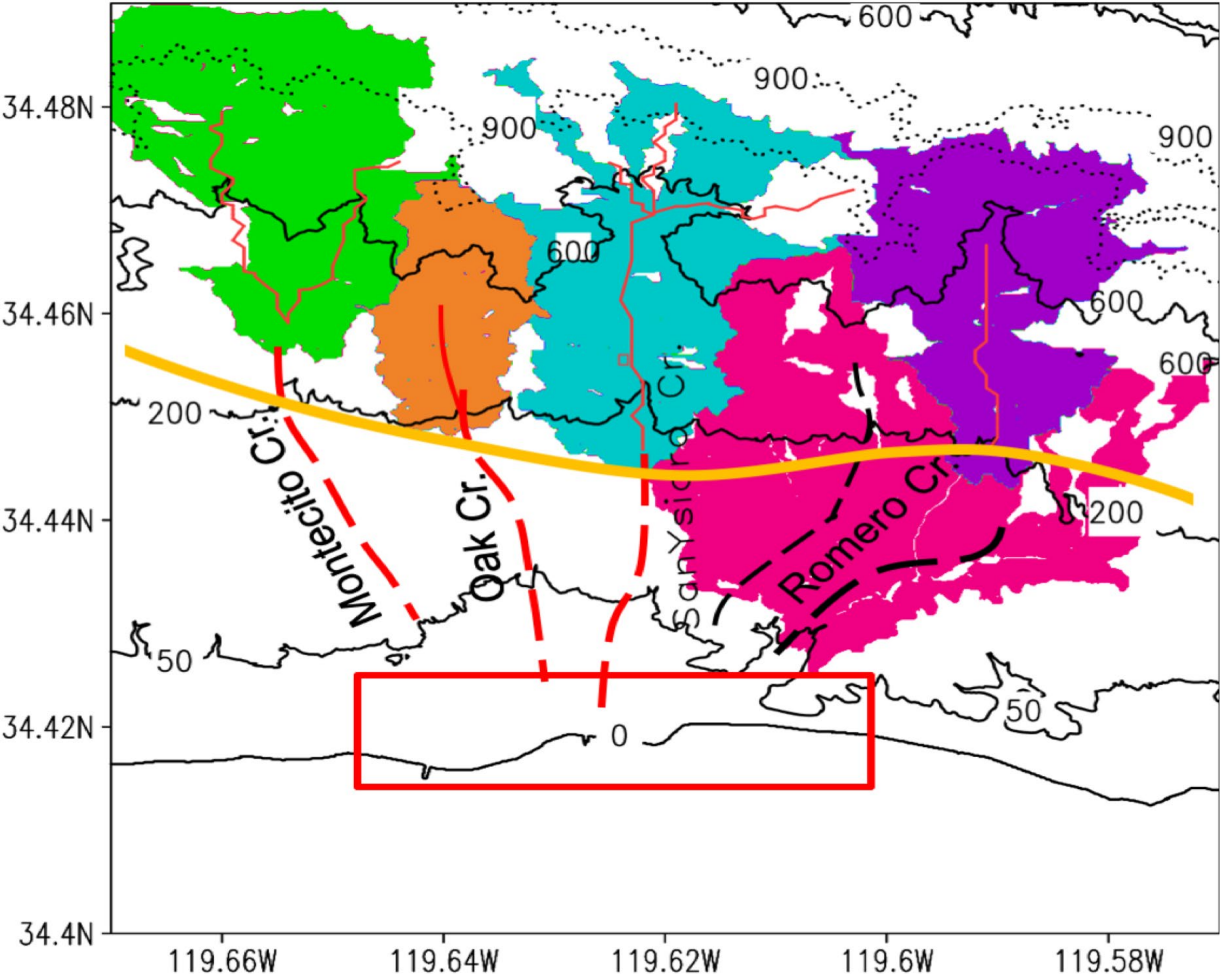
The region of interest. Contours are surface elevation (m). Four major creeks and their collecting basins are labelled. Creeks in the residential area (i.e., south of the bold yellow demarcation line) are dashed. Residential areas are assumed to be impervious (i.e., zero infiltration and contribute no sliding material). About 70% of the slope area has a descending pathway to the depositing region (red-box highlighted area). Avoiding residential encroachment into the floodplain clearly is a priority for disaster mitigation.

During 2012–2017, southern California experienced an extended drought^1–6^. The winter of 2017 (the wet season for its winter-wet Mediterranean climate) experienced unseasonal wildfires, amplified by strong Santa Ana winds and extended into densely populated Santa Barbara and Ventura counties. The Thomas Fire became the largest wildfire in California’s history, as of December 18, 2017. As the wildfires subsided, storm-triggered landslides, more generally referred to as debris flows^7^, occurred on a southward facing slope of Montecito (Fig. 1). Hereafter, the single term debris flow will be used to refer to the Montecito event. The Montecito debris flows were catastrophic, claiming 23 lives, and costing over US$200 million in property damage and clean up^8^. Montecito is at the intersection of numerous factors responsible for debris flows, including climate warming, drought, wildfires, seasonal storms, biological processes, and ongoing tectonic activity^9–11^. Consequently, debris flows such as that of January 9, 2018 are not unexpected.

Southern California is susceptible to debris flows^12,13^, partly because its winter wet season (November–April) encourages plant growth. If the following dry season (May–October) extends into the wet season and strong winds occur, the soil and vegetation both dry out. Sloping terrain then becomes sufficiently unstable for debris flows to be triggered even by less intense storms in the ensuing wet season^14,15^. If there are strong wind events, as has been the case for the past three years, 2017, 2018 and currently in 2019, wildfires further reduce slope stability through multiple mechanisms^7,9,14–20^. Wildfires generate debris that is fuel for ensuing debris flows, as the upper 3-cm of root systems can be totally burnt, while the deeper roots remaining intact. The root web, after entrainment into the debris stream, acts as a nexus organizing the granular particles. Although the Montecitodebris flows were not unexpected^21^, their magnitudes are not yet fully understood. Towards that end, a mechanism contributing to the observed enhanced sediment detachment capability of the debris torrent, is assessed by using the Scalable, Extensible Geofluid Model for ENvironmental Tasks (SEGMENT-Landslide), a 3-D dynamical modelling system^14,15,22^. With a positive feedback loop, the mixture of the sliding (‘sliding’ here is used as a general term for flowing, rolling and other forms of dislocation at granular scales) material, upon reaching lower elevations, entrains coarse granular material (GM) of increasing size, and even scours large boulders. The unusually large magnitude of the Montecito debris flows thereby is explicable.

Section 2 provides a brief description of SEGMENT-Landslide, with further details in the Supplementary Material (SM). Section 3 presents an example of the enhancement effects of the wildfire burning, through organic–inorganic GM interaction, on the Montecito debris flow. Debris flow deposition is analysed and compared with in situ observations. Knowledge gaps are identified for some observed features of the Montecito debris flows, preventing the model from predicting precise occurrence dates of these catastrophic events. These gaps are identified and future research directions are proposed. Section 4 addresses debris flow mitigation, focusing on proactive debris management, especially after major wildfires.

## Method and data

Owing to the highly transient nature of debris flows, direct, first-hand pictures are lacking, especially during the early developing stages. Consequently, a full 3-D process modelling system assumes a unique position in identifying enhancing mechanisms of debris flow. Next is a brief description of the numerical model used and the necessary input data.

### The landslide modelling system: SEGMENT-landslide

SEGMENT-Landslide is a fully parallel numerical code applicable to multiple composition. It is a three-dimensional (3D) model, with high horizontal resolution to represent complex topography in a regional area, making it suitable for a distributed-source area and/or runoff initiation, including burned-area debris flows. As described fully in the SM, SEGMENT-Landslide is a unified approach to several seemingly disparate types of landslides and debris flows. Debris flows can trigger switches for parameterizing entrainment and erosion processes that are not activated for rotational landslides. However, SEGMENT has advanced design features for processing the physical processes within and between grids. These include allowing material within a grid cell to be a mixture of water, GM of various particle sizes, and organic debris from incomplete fire burning; allowing multiple phases; and permitting multiple rheologies of sliding material distributed arbitrarily on slopes. When required, this multiphase, multiple composition capability can be applied to heat transfer, flow turbulence, and fluid–structure interactions. Mechanical properties such as viscosity are parameterized for pure material and also for mixtures, using formulae applicable to a wide range of temperature and pressure variations. The multi-physics capability of SEGMENT-Landslide is highly relevant to debris flow simulations, and is described fully in the SM. Details of the rilling parameterization also are presented in the SM, because overland flow entrainment and rilling are the starting points for the exceptionally large magnitude of the Montecito debris flows. Debris detachment by overland flow primarily is through runoff turbulence^23^, because the energy source for detaching and agitating is the flow shear. For mechanistic modelling of debris flows, the fluctuation–dissipation particle deposition and agitation processes are parameterized using flow shear and mechanical properties of the involved material. Here, a fixed simulation domain (Fig. S1) is used, confining the possible moving material for the entire start-spread-cessation/deposit lifecycle.

### SEGMENT-landslide input data

The required input parameters include static data such as topography, land use, land cover and biomass loading, and related root system properties, as well as dynamic data such as atmospheric parameters. As a subcategory of landslides, debris flows are gravity-driven along slope movements. Uneven surface topography is the ultimate driver of debris motion. Digital elevation models (DEMs) are required as input to the numerical model. The ~ 10 m resolution (1/9 Arc second) DEMs are downloaded from the USGS website (https://www.usgs.gov/core-science-systems/ngp/tnm-delivery/). Data sources and methods to generate the geological setting (e.g., bedrock type and mechanical properties), biological parameters, and initial soil depths are detailed in the SM. The above-ground vegetation properties and fire-scars are primarily remote sensing products, whereas under-ground root system properties are inversely obtained from biomass allometry. Upper limits of soil depths are set up in reference to SHALSTAB, a digital terrain model for mapping shallow landslide potential^24,25^.

SEGMENT-Landslide requires all atmospheric parameters typically necessary for a land-surface scheme. To reproduce the Montecito debris flows, simulations from the non-hydrostatic Weather Research and Forecasting model in its Advanced Research and Forecasting model form (WRF-ARW, V3)^26^ is used. For future projections, atmospheric parameters are provided by climate models. The climate models provide daily precipitation records in the control period comparable to the Global Historical climatology Network^27,28^. Knowledge of precipitation characteristics of southern California also places the Montecito debris flows in their appropriate spatio-temporal context.

### Observed and climate model simulated precipitation

What distinguishes dense debris flows from flash-floods is that the former are not caused by a single variable, or by extreme precipitation. Typically, they involve a complex interaction between several variables, occurring either in sequence or concurrently. Understanding how, when and where the factors combine and interact is critical in providing early warnings, anticipating the occurrence location, planning for, and mitigating the impacts of this weather-related, high-impact hazard. Future precipitation morphology changes play a critical role in understanding of future debris flows for regions of interest. Fortunately, the Californian region has high quality daily rainfall observations at closely spaced GHCN stations. Figure S2 shows the locations of these stations in a 2° × 4° area around Montecito. Table S1 lists the characteristics of 43 GHCN-D stations. Most stations cover January 1, 1981–December 31, 2010, except when explicitly labelled. Due to orography, precipitation over the region is very uneven. To select stations representative of Montecito precipitation, three zones are delineated and this is useful because the stations have consistent recording of daily precipitation (Fig. S3), differing only in magnitudes. The 28 stations in Zone *I* are in bold italics in Table S1. Distances to Montecito are used by the Cressman interpolation scheme to obtain a representative mean precipitation from the 28 stations comparable with climate model simulated rainfall time series.

Future projections of debris flows relevant to climate warming impact are examined from extreme precipitation, simulated by climate models in the Coupled Model Intercomparison Project Phase 5 (CMIP5)^29^ under the Representative Concentration Pathway 8.5 (RCP8.5) scenario. This strong emissions scenario is selected to illustrate possible rainfall morphology changes under a warming climate. The 24 climate models (Table S2) all satisfactorily simulated the twentieth Century southern Californian precipitation statistics.

## Results

To reproduce the Montecito debris flow of January 9, 2018, the simulation domain for SEGMENT-Landslide is a limited, ~ 10 km wide, area covering the region 34.4°–34.49° N and 119.67°–119.57° W (Fig. 1). This basin-scale area contains, from west to east, the Montecito, Oak, San Ysidro and Remora creeks, which contributed most to the catastrophic Montecito debris flows. It is highly populated (Fig. 1), with residential zones extending to 250 m elevation, accounting for ~ 40% of the total basin area. Residential zones are assumed to be impermeable, or without water infiltration and also not contributing to sliding material locally (in contrast to the fire-burnt slopes, where litter can be entrained). The first set of experiments assess how well SEGMENT-Landslide simulates the 2018 debris flows. Assuming a uniform rainfall total of 100 mm in 24 h scenario, potentially unstable valley mouths are identified. Because debris flows are precipitation-sensitive natural hazards, rainfall for a 4° × 5° regional area, around Montecito, are analysed using 30-year observational data from high quality stations. Rainfall morphological changes also are investigated, using 24 climate models with demonstrated skill in simulating current precipitation statistics over southern California^7^. Based on their twenty-first century simulations of atmospheric parameters (from the CMIP5 model archives), locations prone to future debris flows are projected for the moderate, and possibly more likely to be realized, emission scenario RCP4.5. The expected increased frequency of southern California dry rainy seasons, punctuated by increased heavy rainy season precipitation events^2,15^ underlines the enhanced risk of catastrophic debris flows^7^.

### Progressive bulking shallow landslides

Overland flow cuts rills that carry sediment and water to channels, where they gained bulk and cleared/scoured the canyons to bare bedrock, carrying large, destructive boulders into Montecito. Progressive bulking^30,31^ was realized through the upward, extended channelling branches of a converging collecting basin. Figure S4b is a schematic of the processes. SEGMENT-Landslide can simulate all processes: routing of water, cutting rills, gaining bulk from bank erosion sediment, and eventually stalling at the valley mouths.

**Figure 2:**
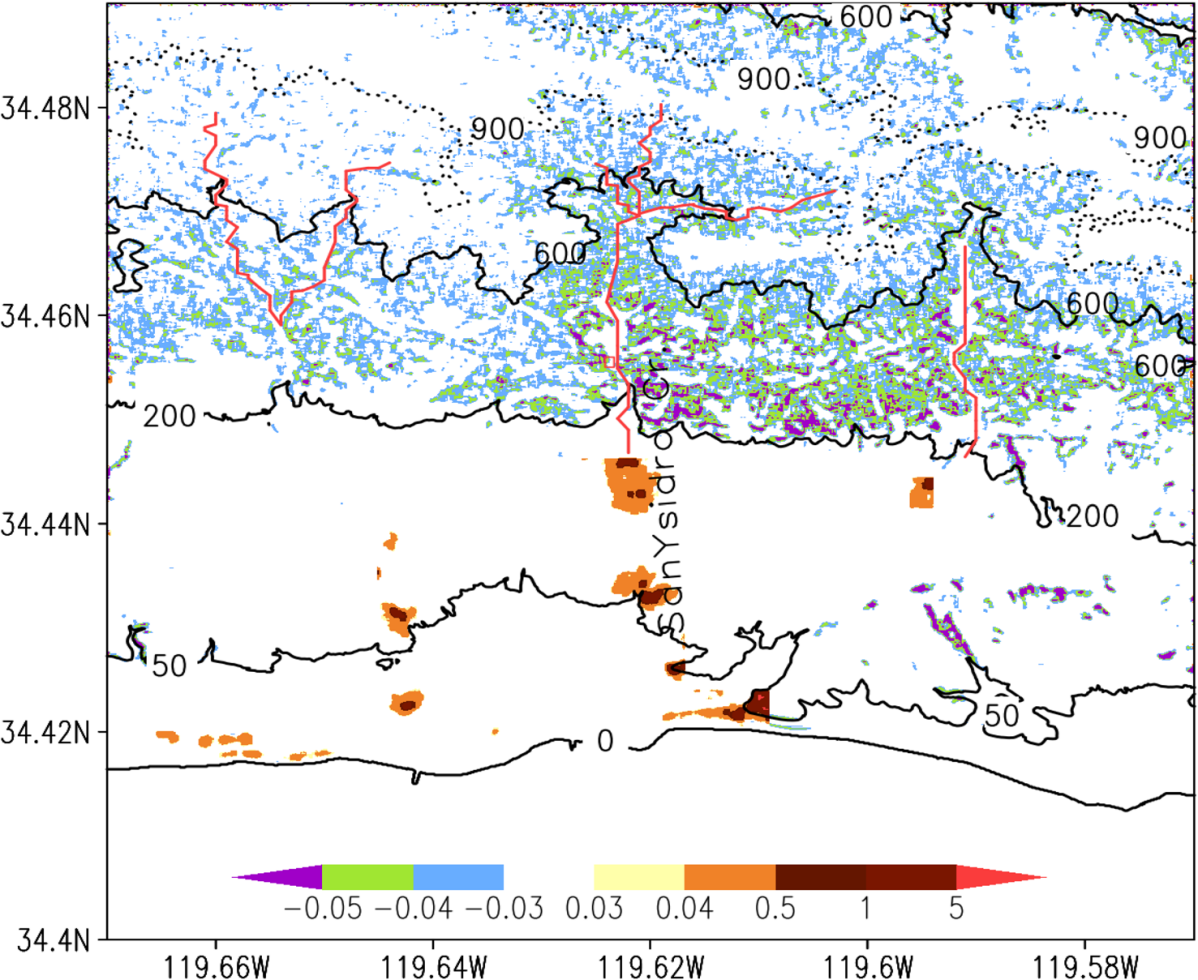
Simulated January 9, 2018 landslides over the Montecito collection basin using precipitation and other related atmospheric parameters obtained from the WRF model. Color shades are changes in surface elevation (in meters) after debris flow ceased (i.e., final configuration minus original DEMs). Most slopes suffered losses of < 3 cm depth of top soils. Only near the valley banks and at relatively lower elevation, significant (> 5 cm) soil erosion occurred. The sliding material, through gullies and creeks, eventually accumulated at the lower, gentle slopes. In addition, ~ 1758 m^3^ of the debris was lost directly to the ocean.

The following are results from SEGMENT-Landslide simulations of the January 9, 2018 Montecito debris flow. Surface elevation changes, representing the source and ultimate destination of sliding material, are in Fig. 2. Above ~ 600 m (the steeper portions of the slope), the debris swept by the overland flow is uniform but small, and not concentrated in significant channels. At lower elevations, erosion is more concentrated. Many channels had no significant net accumulation or mass loss, serving only as debris passages. The storms moved less than 3 cm of topsoil from most slopes. Significant mass loss occurred only near creeks or gully banks (up to 20 cm at places). The percentage of coarse (granular size > 20 cm) sliding material indicates that most gully areas, which contributed coarse GM (and boulders), were refilled with finer grained sliding material so net elevation changes are minimal. The main contribution of boulders to the depositing area is from elevations < 300 m, immediately above the residential area (Fig. 1), and primarily from the San Ysidro Creek and the Montecito creek. Many of the simulated features cannot be verified directly but recent surveys confirmed the generic patterns of debris flow deposition. Based on a very recent study^8^, there are four sediment-retention basins built in 1960’s (Cold Spring and San Ysidro), 1970’s (Romero) and at the turn of twenty-first century (Montecito). All four debris basins had been ‘cleaned prior to the 2018 Montecito debris-flow event’^8^. Post the 2018 Montecito debris-flow event material excavated from the four debris basins summarize to ~ 5.3 × 10^5^ m^3^, about one third is from the San Ysidro basin. The pattern of debris final distribution agrees qualitatively with our simulation. The elevation reliefs between boulder source regions and residential areas generally are < 100 m. Most boulders accumulate at (34.42° N; 119.61° W), far from source regions. How boulders gain such high momentum and travel such distances is of great interest. Difficulty verifying the simulated results arises mainly from insufficient in situ debris observations^32^, whereas some of the simulated features cannot be observed directly, as they are transient and rapidly modify.

**Figure 3:**
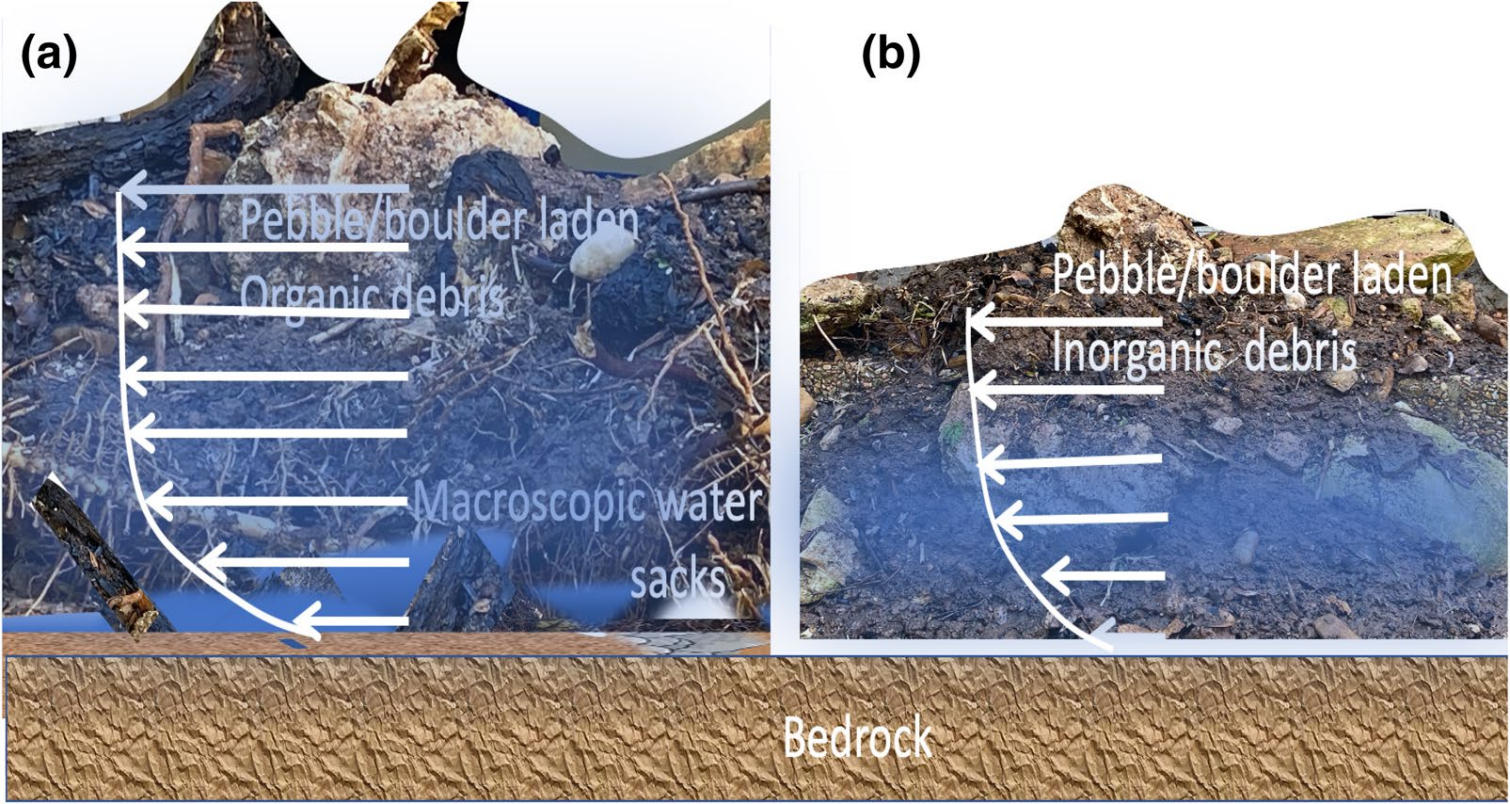
Illustration of the “carrier raft” (**a**), in comparison with inorganic debris (**b**). Boulders resulting from bank erosion fall on top of a web produced by roots entangled with organic debris from incomplete combustion. Because the density of the mixture can be less than that of water, the sliding material actually ‘float’ on top of a water film in the most extreme situation. More generally, the density of the mixture in (**a**) is larger than water but significantly smaller than in (**b**). There will exist macroscopic water pockets at the bottom of the sliding material (also lateral interface with the valley banks). The existence of macroscopic water pockets greatly reduces the bottom and lateral friction of the sliding material when rushing down a valley. This configuration allows a larger debris flow velocity (white arrows) than is possible for similar geometry but when it is composed only of inorganic materials (**b**). This mechanism is rooted in the fact that fire-baked soil and organic granular material have higher water forbicity. Slope-wise, more precipitation is accumulated into runoff (instead of infiltration), gaining increased downstream entrainment capacity.

By monitoring the compositions of sliding/flowing material, a mechanism is posited as being mainly responsible for the unexpected large magnitude of the Montecito debris flows. For the vast areas without apparent scarps, overland sheet flow entrains organic debris produced by wildfires (e.g., stems and branches incompletely burned) and also inorganic debris (loose GM) downslope. Due to the lower density and semi-inviscid nature of the organic clasts, dependency on rainfall amount and intensity to trigger a flow is lowered. The organic clasts move first, and initially entrain inorganic debris of fine grain size. At this stage, there generally is organic debris overlying the inorganic debris. When advected into larger creeks, pebbles and rocks produced by bank erosion can fall onto the organic debris, which ‘floats’ on top of both the water and inorganic debris, due to its lower density. As the mixture gets denser and moves farther downslope an interlaced structure can form, comprising inorganic and organic debris, and pebbles of increasing sizes. The organic debris thereby forms a ‘raft’ carrying the boulders and pebbles. The motion of the sliding material then has greater coherence and greater destructive capacity than individual boulders rolling downhill (Fig. 3a). However, if there is no organic debris from incomplete burning of vegetation, the inorganic debris would be denser and the buoyancy will not be sufficient to support the scoured boulders and carry them in a coordinated manner. Without the lubricating mechanism, the fully developed flows are ~ 4 times lower (Fig. 3b). Despite the latter (configuration) having ~ 120% more mass, the destructive potential is proportional to the velocity cubed, but only linearly proportional to mass, hence the ‘raft’ configuration has two orders of magnitude greater destructivity than the inorganic debris flow. This mechanism, figuratively called “boulder-rafting”, actually is a form of inorganic granular material and organic granular material interaction unique to fire impacted slopes, as discussed in detail in subsection S4 of the Supplementary Material. This mechanism agrees and complements with experimental and theoretical work by Ref.^33^ on granular material segregation, transport and accumulation.

Specific to the San Ysidro Creek, the peak speed of rafts is ~ 12.9 m/s, at about 300 m above sea level (asl). The graded sloping at that elevation greatly reduces flow speeds and causes separation (sorting of GM) of organic and inorganic debris (de-loading of boulders from the ‘raft’). Here, boulders are sorted according by geometry and size, with larger, rounder boulders tending to travel farther, according to the Coulomb yielding criterion. Notably, at this stage, due to increased bottom friction, the debris flows and induced ground trembling are audible at a distance. The total volume of stones involved in this valley alone is ~ 5 × 10^4^ m^3^. Only a fraction (< 10%) of the total reaches flat ground, most stones cease moving in the elevation segment between 260 and 50 m of the creek, to be involved in later debris flows. Below the 300 m elevation segment of the creek is the decelerating portion of the sliding material and the average creeping speed slows to ~ 3.15 m/s, close to eye witness counts^34^ and seismic measurements^32^.

As a concentrated mix of sediments, organic clasts and water, debris flows are advection dominated, fast stages of landscape modification^35,36^. Debris flows can carry large volumes of sediments over extended distances swiftly and powerfully. The unique properties of organic clasts, resulting from incomplete burning of vegetation by wildfires, permits the positive feedback process described above. The mixture of pebbles (and boulders) and organic debris, although possesses a smaller density (than embedding in inorganic debris only), but has significantly reduced friction against bedrock (and also lateral banks). The sliding material thus can reach much larger speed than if they were simply embedded in inorganic granular slurries (Fig. 3). This coherent motion of many boulders has a greater capacity to damage the channels of a graded slope, so larger boulders can be scoured out of the bed and more sliding material then is entrained by the flow. Gravity sorting of debris also assists subsequent debris flows. Like the Montecito case, the slopes rarely are uniform, with rises and steps. For the rafting mechanism, anomalies of < 10 m elevation above the surroundings are no obstacles for the sliding material, if they reside below 450 m asl. This mechanism also enlarges the total sliding material volume reaching the accumulation zone.

A University of California, Santa Barbara research survey noted the boulders were ‘intact’ with no significant damage to 2–3 tonne boulders^33^, because they were agitated not by colliding with peer stones directly but by contact with softer organic components^34^. They were well-cushioned by the organic debris upon entering the flow stream. Thus, the ‘raft’ hypothesis is a physically plausible explanation of the Montecito debris flow. Direct flow measurements at all sliding stages are not available, but when the leading edge of the debris flow reaches residential zones, there are numerous eye-witness reports, photographs and videos available. For the Montecito case, fortunately there was a seismometer ~ 1.6 km southeast of the heavily damaged San Ysidro valley. The ground motion signature of the debris flow was analysed in Ref. ^32^. The debris flow speed near the decelerating stage was ~ 2.5 m s^−1^, at an elevation below 300 m. The rafting stage, with its lower friction against the bed, might be undetectable by current technology. The reported boulder volume of ~ 2,500 m^3^ confirmed our simulation of the boulders reaching the deposition zone. However, there were ~ 6 times as many boulders deposited in the 300–100 m asl segment of the three creeks, and they are a significant threat for upcoming events. The reported duration time also corresponds well with the terminating stage of the simulation. The time to travel from 850 to 300 m, including the early overland flow entrainment stages, took another ~ 20 min. At termination, when the ‘mixed’ debris flow reached the gentle slope at ~ 300 m asl, as a result of gravity sorting, organic debris and finer clasts were trailing boulders. This finding further confirms that a recent observational study of the Montecito debris flow^32^ inversely retrieved just the last stage of the debris flow. Ref.^8^, by analysing similar seismic data, witness reports, and the timing of broken utility lines, provides debris flow speeds at depositing stage close to our simulation. The volume of boulders also is of the same order of magnitude as SEGMENT-Landslide simulated GM in the > 30 cm effective diameter category.

The above experiments, using precipitation close to observed, simulated the debris flow. Because precipitation is patchy, discerning the effects of hydrological features of individual basins on debris concentration, a 100 mm rainfall total is assumed to fall uniformly in a 3-h precipitation window. As debris flows recur over coastal southern California, a larger simulation domain covering (121.2°–117.2° W; 33.3°–35.7° N) is required. Over the Montecito area, the western part of the domain accumulates more debris than simulations with observed/real precipitation. Figure S4b is a representative collecting basin over Carpentaria (119.4°–119.55° W; 34.3°3–34.5° N), adjacent to and east of the Montecito domain. SEGMENT-Landslide was executed over the extended area to identify potentially endangered locations. Examining debris accumulation and the magnitude of destruction gained during its lifecycle, there are ~ 80 locations susceptible to catastrophic debris flows, depending upon precipitation. Locations threatened by debris flows receiving 100 mm of precipitation within three hours are listed in Table S3. Not all have the same possibility of realization, due to uneven rainfall distribution over the region, raising the question of which realization is likely in a warmer, near future climate. The answer is of great public concern, especially for those in endangered areas.

The above-discussed mechanisms on fire-burnt slopes are not limited to Montecito debris flows. To demonstrate the enhancement of possible southern California debris flows, a set of sensitivity experiments was performed with the same setting of 100 mm uniform precipitation over the same 2° × 4° area around Montecito. One setting involved switching off the boulder rafting mechanism by assuming no organic debris (i.e. the fire completely removed all above-ground vegetation). Figure S5 illustrates the increase of large debris flows (involving debris volumes greater than 10^5^ m^3^). With the boulder rafting mechanism switched on, the occurrence of large magnitude debris flows almost doubled.

### Extreme rainfall trends in California

Unlike other types of landslides, debris flows are more sensitive to high-intensity and low-frequency rainfall for initiation. Understanding local rainfall morphology is critical for timely warning, adaptation, and mitigation. From Fig. S6, ENSO is decisive in this region’s precipitation. Years 1982, 1997–1998, and 2004 had high rainy season precipitation totals^37^. After removing the ENSO signal, annual wet season rainfall decreased slightly during 1981–2010. However, there remain many extreme events. In situ observations at a slope station (USC00046657, 33.38° N; 116.84° W and 1691.6 m asl as in Fig. S2), show that the rainy season of 1992–1993 recorded > 750 mm precipitation. Present climate models cannot simulate this level of orographic precipitation. Moreover, orographic regional precipitation is very uneven. Rather than examining all climate model grids in the 2° × 4° box of Fig. S2, only grids with over 0.75 area falling into Zone *I* are counted. A weighted-average of those stations provides a representative value.

**Figure 4:**
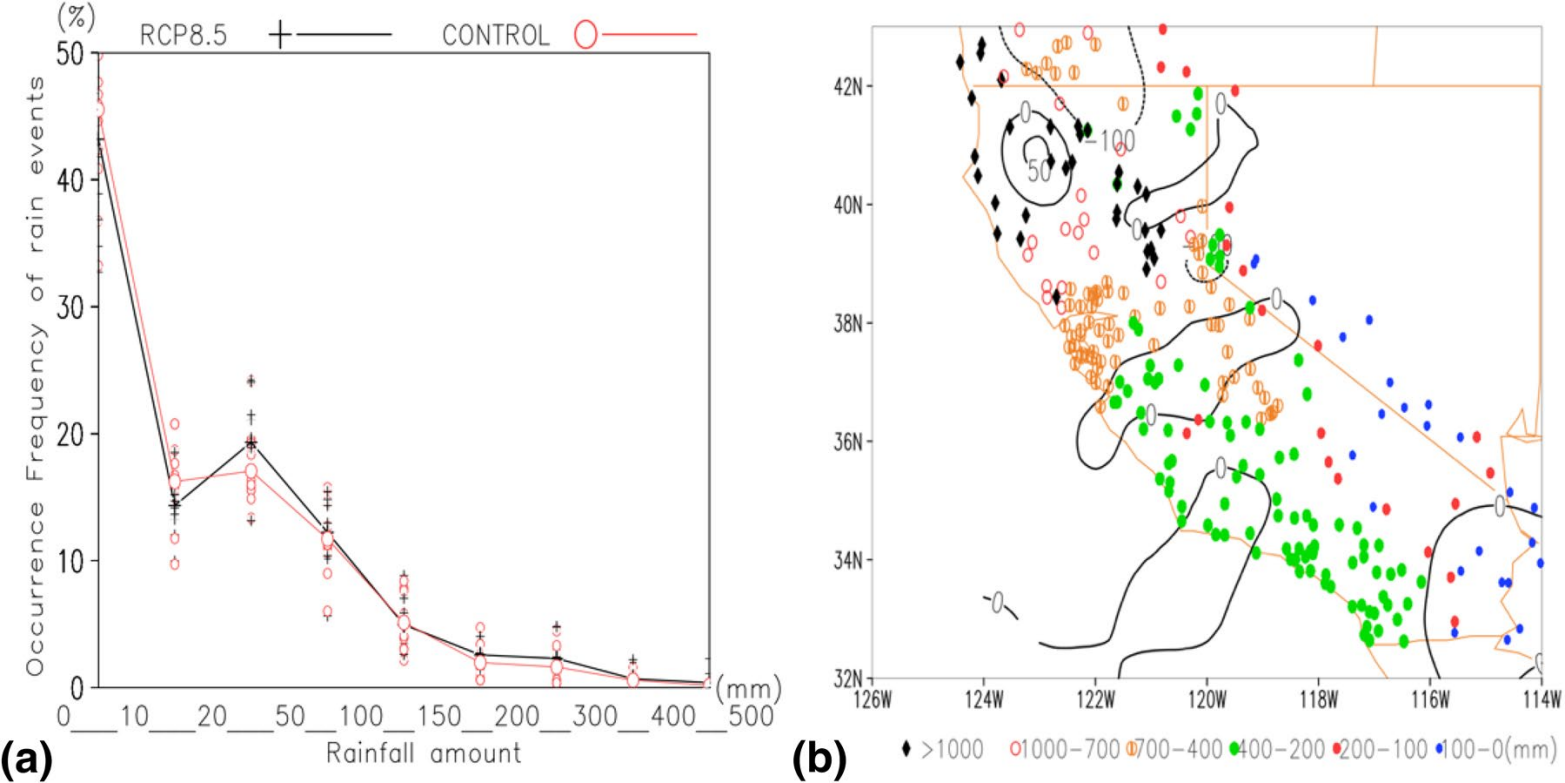
Histograms based on rainfall amounts from rain events occurring in zone *I*, representative of the coastal portion of the region of interest. The present (CONTROL) curve is based on 30-year observations (1981–2010) of rainfall events, with rainfall amounts weight-averaged among the 28-stations within Zone *I*. The RCP 8.5 curve is from climate model simulated rain events with magnitudes scaled according to Li et al.^38^. Climate model grids are used when > 75% of the grid area is in Zone *I*. Shifting into more extreme precipitation is a robust signal for storms producing at least 50 mm total rainfall. For example, over 1.6% of the rain events produce 300 mm totals or greater, compared with only 0.4% of the total storm events reaching this intensity at present. Markers are model spread among the 24-climate models. Panel (**b**) indicates an insignificant reduction in annual total precipitation over Zone *I* (essentially the green dots indicating the 200–400 mm annual rainfall region). Contour lines are annual precipitation differences between the same two periods, as in (**a**): average values of (2,081–2,100) minus those of (1981–2010).

For current GCMs, rainfall seasonality is well-captured^7^, as are spatial patterns, especially in Mediterranean climate regions, due to relatively simple circulation patterns, dominated by subtropical high-pressure systems, and separated by rainfall-bearing cold fronts. Partial removal of vegetation by wildfires, followed by rain storms, caused the failure of the Montecito slopes. Neighbouring regions with similar hydrological features still pose threats to highways and residential neighbourhoods. Knowledge of extreme precipitation changes in a warming climate is critical for mitigating debris flows. Here, for debris flows, rainfall within a single day or spread over several consecutive days, rather than daily values, are used to calculate the following statistics. Using observed rainfall for 1981–2010, and climate simulated rain events for 2,081–2,100, averaged over the 24 CMIP5 models, under RCP 8.5, histograms are shown (Fig. 4) for 9 bins dividing the 0–500 mm rainfall total. Under RCP 8.5, rain event magnitudes simulated by the climate models are scaled^38,39^. Comparing present and future histograms, the rainfall shifts to more intense events under the RCP 8.5 scenario, for all rainfall events exceeding 50 mm. By the late twenty-first century, over 5.8% of rain events are projected to produce 200 mm rainfall, compared with ~ 1.3% at present, in the context of decreasing annual rainfall (Fig. 4b). However, the number of rain events does not change significantly (~ 11.2 storm events annually, not shown), indicating a “drought-intense storm” bipolar pattern^40^. The above estimates should not be taken literally, especially for individual years. The annual number of rainfall events varies from 6 in 1990 to 17 in 2090, which are fluctuations too large for accurate simulations by current climate models. Similarly, a large climate model spread also discourages taking the transition category literally (the two histograms intersect; see the 20–50 mm rain events in Fig. 4a). The value of the climate model simulations is that it identifies the inland shift of rainfall, and suggests heavy precipitation events will be more intense.

Based on estimated precipitation from the 24 climate models, under the moderate RCP 4.5 scenario, SEGMENT-Landslide predicts locations that are susceptible to debris flows, prior to 2050 (bold-italics in Table S3). Due to the bi-polar tendency of changes in precipitation, wildfires are likely to be more frequent in a future warmer climate, as already noted in several very recent studies (Ref.^41^ and references therein). Debris flow could be a consequence of, or at least be enhanced by wildfires, by introducing hydrophobicity conditions that increase the magnitude of runoff from ensuing storms. Due to increases in burning and post-fire hillslope erosion, the sediment yield is expected to increase in the near future (e.g., 2001–2050)^41,42^. This further contributes the enhanced magnitudes of future debris flows. As there are perspectives on changes in future wildfires as the climate warms^43,44^, it is a planned next step of this research to examine the future wildfire occurrence pattern and their effects on slope stability over the entire US and ultimately globally. The simulation presented here provides a clear understanding of the amplification of the magnitude of debris flows involving the wildfire-reduced organic granules. A salient consequence of climate warming is pervasive frequency and severity changes in extreme precipitation^1,2^. Globally, without a significant overall Bowen ratio change, the annual total precipitation balances net outgoing longwave atmospheric radiation. Hence, as the climate warms, global total precipitation should increase. A current difficulty for climate models is to match their regional projections with reality, especially for precipitation because, for different climate zones, local recycled moisture source and remotely advected moisture sources consume different portions^45–47^.

In debris flow research, climate model predictions of extreme precipitation events are challenged by highly localized regional aspects, including orography and small-scale microphysical processes (e.g., the heterogeneity of land surfaces) not explicitly represented by the spatial resolution of climate models. In addition, the definition of extreme precipitation as continuous precipitation over successive days^14^ should be adopted. Such a definition better reflects soil moisture conditions than the current daily rainfall definition. By extracting a climate model consensus of future precipitation statistics in intensity and duration, future research is planned to examine future wildfire occurrence patterns and their effects on slope stability. The debris rafting mechanism identified here will assist understanding of the magnitude of debris flows involving wildfire-reduced organic granules.

## Conclusions

Incomplete burning of vegetation from wildfires produces light, semi-inviscid, organic debris. Its unique physical properties allow a weaker trigger to initiate debris flow and also, more importantly, permit a more powerful and damaging configuration of the organic debris and the pebbles/boulders that it webbed during downslope motion. Hence, a debris flow has higher density boulders embedded in organic debris woven into a ‘raft’ sliding down slopes. The catastrophic Montecito 2018 debris flow simulated here, that occurred in a meteorologically and geologically densely instrumented region, had verifiable features attesting to the validity of the debris rafting hypothesis. This debris rafting mechanism underscores the critical role of debris management in the adaptation and mitigation measures.

The southern California Mediterranean climate triad of fire, extreme rainfall and devastating debris flows frequently threatens the welfare of residents. The recent case of the Montecito debris flows following an extended drought (2012–2015) and the largest wildfire in California’s history (the December 2017 Thomas Fire) bespeaks the pattern would be a new normal in a warming climate. In a warming future, there is an expected intensification of the existing bi-polar drought-rainfall mode. The debris rafting mechanism suggested here (illustrated in Fig. 3) describes how such a warming climate can amplify the destructiveness of debris flows. Reliable, mechanistic physical models are becoming available to predict this type of natural hazard. They can provide timely warning so that loss of property and life can be reduced in the big data era.

The very fact that there are casualties of the Montecito debris flows bespeaks of the inadequacy of the current mitigation and adaptation system employed. The framework of the current most successful advanced warning systems for debris flows, the joint NOAA-USGS endeavor (e.g., NOAA-USGS Debris Flow Task Force 2005) could significantly reduce the false-alarming rate if a mechanistic physical core such as SEGMENT-Landslide is used instead of the empirical rainfall-intensity duration scheme. This is because SEGMENT-Landslide can simulate the entire start-spread-stop cycle of debris flows. The Montecito debris flows underline the need for improved proactive mitigation and adaptation policies. Much of the value of the debris flow models, such as SEGMENT-Landslide, is due to their capacity to increase the probability of early detection, while reducing the false alarm rate to minimize evacuation fatigue.

## Supplementary information

Supplementary file
