# Supplementary material for: Climate warming enhancement of catastrophic southern California debris flows
Source: Sci Rep. 2020 Jun 29;10:10507. doi: 10.1038/s41598-020-67511-7 (PMC7324592; doi:10.1038/s41598-020-67511-7)
Supplement: Supplementary file 1 — Supplementary file [file 41598_2020_67511_MOESM1_ESM.docx]

**Climate Warming Enhancement of Catastrophic Southern California Debris Flows**

Diandong Ren^1,2^ and Lance M Leslie^3^

1. School of Electrical Engineering, Computing and Mathematical Sciences, Curtin University, Perth, Australia
2. College of Oceanography, Hohai University, Nanjing, China
3. School of Mathematical and Physical Sciences, University Technology Sydney, Sydney, Australia

Submitted to Scientific Reports on September 24, 2019

Revised on April 16, 2020

**Corresponding Author Address:** [Diandong.ren@hhu.edu.cn](mailto:Diandong.ren@hhu.edu.cn); [Diandong.Ren@curtin.edu.au](mailto:Diandong.Ren@curtin.edu.au)

**Supplementary Material (SM): SEGMENT-Landslide, an extensively tested landslide modelling system**

SEGMENT-Landslide provides direct information of the hazard's profile (i.e., magnitude, frequency, duration, presence timing, and spatial extent), rather than atmospheric conditions as proxy for landslides, such as intensity-duration methods. After a decade-long evolution, SEGMENT-Landslide now is a fully parallelized numerical code, handling multiple compositions, multiple phases (e.g., the co-existence of two or more immiscible, incompressible fluids), and multiple rheologies of the moving material distributed arbitrarily on inclined terrain. Multiple-composition means that materials at a grid point are allowed to be a mixture of water, granular material (GM) of various particle sizes, density contrasts, internal frictional angles and associated mechanical properties. It also permits the presence of organic debris (e.g., from incomplete fire burning), and bedrock that is stationary, and provides the lower boundary conditions for the model simulation. Multiple-rheology requires that the mechanical properties of the materials, especially viscosity, are parameterized using formulae applicable for a wide range of temperature and pressure variations.

Highly relevant to landslides and debris flow simulations is the multi-physics capability of SEGMENT-Landslide. With its treatment of flow-rigid boundary interactions, simulating transient flows is a major strength of SEGMENT-Landslide. For debris flows, a fixed simulation domain (Fig. S1), which encompasses all of the possible moving material over the entire lifecycle of start-spread-cessation (deposit), is used. The following section discusses the unique design philosophy, grid stencils and parallelism strategy, together with the presentation of the governing physical equations and, finally, the execution procedure. The physical phenomenon is that of a dynamically and spatially varying water flow with entrained debris. The detaching and agitating, transporting, and depositing of the flowing material in the valleys (a generic term used here for rills, gullies henceforth) all are simulated by the model.

**S1. The grid stencil and parallelization strategy**

SEGMENT-Landslide uses a terrain-following, vertically stretched σ-coordinate stencil to better represent the upper material interface of the slope material (Fig. S1a). The horizontal grids are very fine latitude/longitude grids, to fully utilize the 10 m spacing of the DEM. An Arakawa C stencil is applied (Fig. S1b) so that scalars (*S*) and the vertical velocity (*w*) project to the same latitude/longitude location and are surrounded by horizontal flow fields (*u* and *V*). Potential sliding materials are well-confined in this simulation domain, during the entire collecting-sliding-spread-cessation lifecycle. Material at a grid point is allowed to be a mixture of water, GM of various particle sizes, including possible organic debris from incomplete fire burning. SEGMENT-Landslide supports multiple rheology of the medium it simulates. In idealized model verification runs, its multiple flow simulation capability has been extensively tested (not published but available per request). For example, the co-existence of two or more immiscible, incompressible fluids is well-represented. The multi-phase, multiple- composition capability may be used for heat transfer, turbulence, and fluid-structure interactions. SEGMENT-Landslide also has a large set of non-conformal and conformal grid stencils available for a wide range of applications. For the debris flow simulations in this study, a fixed simulation domain, as described above, is used that confines the possible moving material throughout the flow event.

The SEGMENT-Landslide code is designed to run very efficiently on supercomputers. It has horizontal domain parallelism using halos of width 2 (grids), with physical processes parallelized. Hence, each increase in the number of processing nodes will further increase the system’s efficiency.

**S2. Governing equations**

Of key relevance to landslide and debris flow simulations is the multi-physics capability of SEGMENT-Landslide, especially the mechanical properties of the materials. Density, particle size, the internal frictional angle, and viscosity are parameterized for pure materials and their respective mixtures, using formulae applicable for a wide range of temperature and pressure variations. There are no limitations on the density ratio, viscosity ratio and triple-point ratio that are typically required by many current, comparable commercial software systems. With novel treatment of the flow-rigid boundary interactions, simulating transient flows is a major strength of SEGMENT-Landslide. The following sub-sections outline the governing equations for the generic framework of SEGMENT-Landslide, with emphasis on parameterizing granular agitation and entraining processes into governing equations.

***S2.1 The momentum equations***

The momentum equations, with parameterized viscosity, namely,

$\rho\left[ \frac{\partial\vec{V}}{\partial t}+\nabla\cdot\left( \vec{V}\otimes\vec{V} \right) \right]=\nabla\cdot\Sigma+F$, (S1)

governs the flow regime. Here $\rho$ is the density of the creeping material, $\vec{V}$ is the full 3D velocity vector, $\Sigma$ is the internal stress tensor, and *F* is the body force (e.g., gravity). This equation, with parameterized viscosity, governs the flow. Viscosity, through the constitutive relationship, connects the flow and the stress fields. The constitutive relationship is detailed by Ref. 22. Tensile stress from vegetation roots, the above-ground vegetation loading and soil cohesion all are included in the formulation of the total stress tensor. In parameterizing the vegetation effects, we referred to many research publications, including Refs. 25, 48-54. Biological influences on slope stability are multi-faceted and include formulation of the impact of surface roaming, foraging animals^55^, the burrowing of soil worms and other soil animals, and the secretion of chemicals in strengthening and weakening the bonds between the clasts. These influences are not yet implemented in SEGMENT-Landslide, but will be incorporated in a future version when relevant literature becomes available to provide verification. Currently, SEGMENT-Landslide shows that above-ground vegetation is a stabilizing factor for moderate (less intense) levels of precipitation, through its fortification of the medium by both a distributed root web, and the canopy’s interception of rainfall. When the rainfall is sufficiently intense and exceeds a threshold amount, it soaks the entire soil mantle and all of these preventative effects diminish and the vegetation participates in the sliding, and even increases the magnitude of the event through its particular physical properties (e.g., its lower density than both water and the granular inorganic particles). The mass conservation equation (details below) uses the flux form to assist in implementing the various external forcing (or source) terms of the composition components.

***S2.2 The thermal equations***

For the sliding material, the kinetic energy gained from lowering the potential energy eventually is dissipated as heat, through internal and boundary friction (e.g. energy cascading). In addition to the fact that viscosity and many other mechanical properties are functions of temperature, the thermal equations are needed also because dynamic soil moisture balance in SEGMENT-Landslide requires this parameter (detailed below). The relevant equation is:

$\rho\left[ \frac{\partial T}{\partial t}+(\vec{V}\cdot\nabla)T \right]=\kappa\Delta T+2\sigma_{eff}^{2}/\nu$, (S2)

where $T$ is the temperature of the medium, $\kappa$ is the heat conductivity, *Cp* is the heat capacity, $\nu$ is the viscosity, and subscript *eff* on stress tensor $\sigma$ means effective. The forcing terms on the right-hand-side of Eq. (S2) are respectively heat diffusion and internal heating. The effective operator is defined as $2*_{eff}^{2}=*_{xx}^{2}+*_{yy}^{2}+*_{zz}^{2}+2(*_{xy}^{2}+*_{xz}^{2}+*_{yz}^{2})$ ,where the asterisk stands for any quantity to be operated on. Effective stain rate, which is used in the parameterization of viscosity $\nu$, is formally defined similarly. Viscosity, for different composition is defined very differently. For coarse GM, see Ref 22.

SEGMENT-Landslide has one liquefaction mechanism which is related to intense evaporation. The friction (both internal friction and friction against the bedrock) due to sliding can easily raise the material’s temperature, sometimes exceeding 100°C. Intense evaporation within the porous material forms bubbles within the sliding material, especially between bedrock and the overlying sliding material. The overlying sliding material therefore acts like a “hovercraft” floating on air cushions. The following paragraph describes how this process is parameterized in SEGMENT-Landslide.

Air bubbles within the sliding material near the bottom resulting from intense heating are calculated using the temperature profile from the land surface model component of SEGMENT-Landslide. The convergence of heat flux plus the internal strain heating provide the energy for vaporization. Aside from the canonical parameterization of heat transfer quantities (e.g., conductivity and diffusivity), for the porous media involved, the thermal conductivity is parameterized in SEGMENT-Landslide as,

$\kappa_{e}=\kappa_{w}\frac{\varphi\kappa_{w}+(1.5-\varphi)\kappa_{G}}{(1.5-0.5\varphi)\kappa_{w}+0.5\varphi\kappa_{G}}$, (S3)

where $\kappa_{e}$ is effective thermal conductivity of water under the confining pressure and temperature environments (e.g., order of 3 atm and 80 ºC). Subscripts ‘*w*’ and ‘*G*’ denote respectively the water and the granular materials, $\varphi$is the porosity. This formulation is based on the molecular and turbulent processes of aeration that superimpose effects on thermal conductivity. In this formula, liquids are a larger proportion of the two-phase mixture in determining the overall thermal conductivity. The correct asymptotic limits are reached (i.e., for fully solid rock or pure water, it reaches the conductivities respectively of rock or water).

Once aerated, the cohesion (in the Gibbs energy bundle) is diminished and the aeration effects on viscosity are parameterized as a factor, *C_a_*, multiplied by the moisture impact on the kinematic viscosity of the granular material as:

$C_{a}=(1+r_{0}/r)^{-0.5}$, (S4)

where *r_0_* is the radius of stable bubbles inside pure water under the same pressure and temperature environments and *r* is the ensemble size of the GM. This admittedly is more commonly applicable for circular scarp landslides than debris flows. However, the convex basin, working together with graded sloping in the Carpentaria region fostered such instances in the 2018 case (such a shear zone occurred after the flow becomes unsaturated, as a result of active entrainment, at elevations ~320 m above sea level).

***S2.3 The continuity equation***

SEGMENT-Landslide follows a continuum approach. From the continuity equation, flux convergence (divergence) is the primary mechanism for the bulking (thinning) of sliding material. For any trace flow component of sliding material of volumetric concentration *S* (a scalar quantity),

$\frac{\partial S}{\partial t}+\nabla\cdot(S\vec{V})=$*F_E_*, (S5)

where *F_E_* is the volumetric concentration of the source/sink term (i.e., from the entrainment scheme). Physically, the flux divergence/convergence is the dynamical cause of concentration fluctuations of the quantity of interest. The forcing (source) term has different interpretations for different components. For example, for water content in the sliding material, instantaneous precipitation is an important source. The evaporation could be a sink once the sliding material is heated by both internal friction and friction against the lower boundary. For granular material, the source could be from the detachment of gully bank and rill erosion. For organic debris, the forcing term is minimal and is essentially a redistribution by advection.

Equation (S5) sufficiently explains the slope-dependent transport of debris, accrued from weathering and other erosional processes. That is, any valley, hollow, swale, or other even subtle indentation into the hillside will be a site where soil transport will converge and cause net soil accumulation if there is no channel present to effectively remove the converging soil. Graded sloping evolves as a result of the basic requirements of mass balance and lowering of potential energy of the system. At longer time scales, the thickness of the soil mantle also is controlled by the flow divergence and convergence (i.e. valleys possess deeper soil and ridges possess thinner soil thickness). In setting up the soil thickness, the slope-dependent assumption of Ref. 25 is followed. This prescribes an upper limit on how much soil can be eroded by debris flows. Typically for landslide areas, there is a clear boundary between the soil (i.e., the O, A and B soil horizons in the USDA soil taxonomy) and the underlying variably weathered bedrock. Mass concentration of coarser grained GM and boulders are estimated from geological maps of the region of interest, assuming a sedimentation rock formation during low energy state (i.e., coarser material increases with depth in the ground). In this aspect, SEGMENT-Landslide shares the SHALSTAB^24,25^ perception of the potential sliding material. The essence in the treatment is with viscosity and mechanical properties of the moving material. Debris, either the organic components as a result of incomplete burning or the inorganic GM, and the involved fluids are parameterized by their mechanical properties. As a finite difference model, SEGMENT-Landslide does not follow the moving of the flow elements but instead their ensemble (gross) properties are parameterized. Each spatial grid possesses a set of such parameters and they vary as time marching progresses and the processes evolve. The continuity, energy and momentum equations are solved simultaneously so the required parameters for entrainment, detachment/scouring of debris on the slope are readily available.

For this study, the runoff concentration follows existing topographic features (gullies and creeks) discernible from the 10-m resolution digital elevation map. Finer scale rilling processes are not directly simulated but the overall effects of soil erosion are parameterized using the flow shear, the bed slope, lateral bank slope and their composing materials’ mechanical properties. Specifically, in SEGMENT-Landslide, effective strain rate (${\dot{\text{ε}}}_{eff}$) is used as a criterion of whether or not a detachment is possible:

${\dot{\text{ε}}}_{eff}>E/\nu$ (S6)

where *E* is bedrock/bank tensile strength against peeling, $\nu$ is the viscosity of sliding material, and ${\dot{\text{ε}}}_{eff}$is the strain rate at the interface of sliding material against rill beds or gully banks. The effective operator is defined as $2*_{eff}^{2}=*_{xx}^{2}+*_{yy}^{2}+*_{zz}^{2}+2(*_{xy}^{2}+*_{xz}^{2}+*_{yz}^{2})$. For multi-composition, multiple rheology SEGMENT-Landslide, deposition is an internal process, or mass exchange among adjacent grids. The detachment (scouring) and deposition are independent processes. For a wide range of flow regimes, there is neither deposition nor detachment, consistent with the fact that debris transportation is the primary function of a channelling system. Equation (S6) governs when detachment occurs. The actual sediment entrainment rate is given by:

$F_{E}={(\rho}_{l}C_{D}{\dot{\text{ε}}}_{eff}\left| \vec{V} \right|/J_{0})d\rho_{s}$ (S7)

where *F_E_* is volumetric concentration of source, $\rho_{l}$ is density of the sliding material, *C_D_* is drag coefficient (proportional to viscosity), $\left| \vec{V} \right|$is flow magnitude at the boundary, for bank erosion, $J_{0}$is energy for fatigue dislocation, *d* is granular diameter, and $\rho_{s}$ is bedrock density. From energy consideration, there are two steps involved: detaching and agitating, both originating at the interface of flow and solid boundary. Flow shear is the energy source for both the laminar stage and turbulent stage, with detached eddies as the “hammer” to detach and agitate. The energy required for detaching particles is the product of cohesion stress, cross-sectional area, and the molecular dimension of the composition material. Agitating of the detached particles are parameterized, following Ref. 56, for energy expended on transport of particles, with flow discharge rate replaced by kinematic viscosity. In mountainous regions, the ease of peeling material from river banks and gully beds is not constant because it becomes progressively more difficult as erosion continues. This primarily is due to the fact that the weathering starts from the surface (the interface with air) and the degree of fracturing decreases exponentially deep into the solid medium. Thus, the stream power is further modified by a factor indicating the degree of weathering. This factor decreases exponentially as erosion (entrainment) progresses. With this parameterization, material can either be deposited mid-slope, delivered down the slope with little added material from the creek beds and banks, or be magnified by the added material from bank failure and bed scouring. Slope morphology thus is an integral component of the SEGMENT-Landslide modelling system.

**S3. Bank erosion (collapsing) and rilling processes**

It is widely agreed that bank erosion and rilling are important processes in debris flow bulking^23^. The following parameterization scheme relates the flow shear to the material generation rates. With the Eqs. (S1), (S2), and (S5), SEGMENT-Landslide provides surface elevation changes before and after the sweeping of the slopes by storms, under the circumstances where shear dominates the entrainment capacity. Net gain or loss of the sliding/moving material is determined by the competing processes of entrainment and deposition rates. Sedimentation deposition rate also is diagnosed formally following the same flux equation, with the scalar property expressed now in the form of sediment concentration, *c*. The corresponding forcing term now involves diffusion, namely $\nabla(A*C_{D}*\nabla c$), with A the areal element of integration and C*_D_* the dispersion coefficient (or the diffusion coefficient) in m^2^s^-1^. The forcing term also involves the mass gain from bank erosion or mass loss as deposition, parameterized using flow features corresponding to the potential transport capacity^56^. A shear versus hardness criterion is used instead (see Eq. S6), to avoid detachment and deposition being the only two options without intermediate stages. This treatment is based on the basic fact that the usual function of a valley is transporting the sliding material.

Through these interlinked processes, a loop is established between the flow and the gullies that channel the sediment flow down the slope. Whether the gulley gets deeper, wider or is filled with debris is dynamical, depends on the instantaneous stress conditions. Compared with many simplified treatments of local slope stability, SEGMENT-Landslide, by simulating the interlinked processes, indicates that it is not just the local slope that matters, but also the curvature of the topography and how that feature concentrates or diverges runoff downslope. The entire lifecycle from start through spread and then cessation of an event is simulated in a physically consistent manner that conserves energy, momentum and mass.

**S4. Fire effects on slope soil and vegetation**

To obtain a set of parameterization schemes that are applicable across multiple soil-types and fire regimes, we have cross-verified with laboratory results^57^, *in situ* intact sample measurements^58^ and field measurements indicating non-uniform dependence of repellence with respect to burning severity^59^.

S4.1 Fire effects on soil physical properties

Effects of wildfires on slope soil and vegetation clearly are important for the Montecito case. From the governing equations, post-fire changes in soil physical and hydraulic properties are critical for controlling the magnitude of the debris flows through the impacts on runoff and infiltration partition. Soil physical properties directly used in SEGMENT-landslide include bulk density, dry repose angle, cohesion, and saturated hydraulic conductivity. Soil organic matter content and water repellence directly affect soil density and hydraulic conductivity.

SEGMENT-Landslide, as stated above, permits multiple composition in granular material. Slope soils are classified into organic granules and inorganic soils. During incomplete burning, fire reduces aboveground vegetation into organic granules (e.g. duffs and litters) that are mixed with inorganic soils during debris flow initialization and development. The total amount of organic granules ($M_{o}$) from aboveground vegetation burning is parameterized as

$M_{o}=M_{v}\Upsilon(1-{0.002\left( B-B_{0} \right)}^{2})$, (S8)

where $M_{v}$is aboveground biomass density (kg/m^2^), $\Upsilon$ is the canopy allocation ration (roughly the vegetation material that can be burnt and fall to ground; or the upper limit of fire-reduced organic material from vegetation), *B* is the burning severity classification as in the Burned Area Reflectance Classification (BARC)^60^, and $B_{0}$is the optimal burn severity that maximally produce organic debris from aboveground vegetation. With this parameterization, too light burning (e.g., low temperature burned areas) and too severe burning (high-temperature burned areas) both reduces the organic debris falling on slopes. A logarithmic bin level is assumed for organic particle size distribution: <0.3 cm, 0.3-1-3-9-27-81, and >81 cm. A uniform distribution among bin levels are assumed. Organic particles of effective diameter size less than 3 mm are considered as soil organic material (e.g., considered in the parameterization of bulk density). With the fire baking and changes in soil composition, the water repellence factor is parameterized as

$f_{R}=1-2\times{10}^{-4}Bb$. (S9)

Where *b* is Clapp and Hornberger pore size distribution index^61^. The use of remotely sensed burn-severity metric *B* in parameterizing wildfire impacts on soil physical and hydrological properties was justified by many recent studies^62,63^. Saturated hydraulic conductivity is parameterized as unburned soil value multiplying this repellent factor.

In SEGMENT-Landslide, effects from wildfire burning decay with time exponentially,

$f_{d}=e^{-ct}$, (S10)

where t is time (in year) and *c* is a constant coefficient (0.33). This seems suitable for light to medium burned slopes. With this set of parameterizations, wildfires lead to enhanced debris flows magnitudes, through increased runoff generation in sloping environments and through the increased organic granules produced by incomplete burning. This is the root mechanism for the boulder-rafting, or boulder-assisting, mechanism.

S4.2 Fire effects on vegetation

The vegetation types and aboveground biomass directly determines the extent to which the organic debris can be reduced by wildfires. For example, the chaparral shrub slopes of southern California and a pine-tree covered Colorado Front Range slope may experience very different surface heating during wildfires. In addition, wildfire removal of aboveground vegetation causes de-loading of slopes and consequently changes to the stress field within the sliding material. The root system caused reinforcement effects deeper than 3 cm are usually not affected by wildfires at the first growing season (e.g., before decaying of the roots; personal communication with T. Dunne 2019). Equations S8-S10 thus are applied to only the upper 10 cm of the slope soil. For the Montecito debris flows, the reduction of root system reinforcement is minimal. Generically in SEGMENT-Landslide, the root tensile strength also decays with time as $\boldsymbol{f}_{\boldsymbol{d}}\boldsymbol{=}\boldsymbol{e}^{\boldsymbol{-ct}}$ (Refs. 64, 65**)**. Therefore, fire occurred outside of a 5-year window of the simulation time is negligible. Vegetation effects are more important at the later stage of debris flow developments. Detailed parameterization of soil mechanical properties is available in Ref.9.

Hence, the mixing of organic debris and inorganic granular material, as the stream rushes down the slopes, results in a mixture of less density (usually still greater than water but much less than 2.7×10^3^kg/m^3^, the density of mineral soils and stones) and thus higher buoyancy (not literally floating on water but possessing higher buoyant mass in the general framework of the diffusion-advection sedimentation theorem). Incompletely burnt surface litters and unburned roots form the sources for organic debris. Thus, ‘boulder-rafting’ or ‘boulder-assisting’ phenomena is more prevalent on wildfire impacted slopes during debris flows.

**S5. Input data to SEGMENT-Landslide**

As a full 3D mechanistic physical modeling system, the performance of SEGMENT-Landslide relies crucially on the input parameters. From the governing equations discussed above, the required input parameters include static ones such as the topography, land use, land cover and biomass loading (and related root system properties), as well as dynamic ones such as atmospheric parameters. The description of the input data will not be inclusive here but instead it will focus on those most relevant for the region of interest.

As a subcategory of landslides, debris flows are gravity-driven along slope movements. Digital elevation models (DEMs) are required as input to the 3D numerical model. The10-m (1/9 Arc second) DEMs are downloaded from the USGS website (<https://www.usgs.gov/core-science-systems/ngp/tnm-delivery/>). SEGMENT-Landslide requires all atmospheric parameters of an usual land-surface scheme. For reproducing the Montecito case, simulations from an advanced weather prediction model, the non-hydrostatic Weather Research and Forecasting model in its Advanced Research and Forecasting model^26^ (WRF-ARW, V3) is used. For future projections, atmospheric parameters provided by climate models are used instead.

Initial field surveys and GIS mapping revealed dense networks of rills on bare, burned hillslopes of the shale formations. Despite similar rainfall and wildfire conditions, rill occurrence was virtually absent from sandstone formations and the most extensive ground disturbances were gullies and shallow colluvial failures. Geological maps of California (<https://mrdata.usgs.giv/geology/state/state.php.state=CA>) are used to differentiate shale and sandstone formation of slope surface. The coarse sandy top soil are weathered primarily from shales and sandstone. The non-weathered sandstone composes boulders embedded in the slope and are scoured out when the situation permits. In SEGMENT-Landslide, the relevant rilling scheme is a flow shear-based parameterization (Eqs. (S6) and (S7)).

Fire scars are obtained from MODIS imagery, using both the monthly global 500 m MCD45A1 burned area data products, which are combined Terra and Aqua satellites, and the 8-day MOD14A2 products, available at <https://e4ftl01.cr.usgs.gov/MOLT/MOD14A2.006/>). A burnt-area mask for the 2018 wildfires is made for coastal southern California. For vegetated surfaces, especially those affected by fires, representing soils as purely mineral is inadequate (Ref. 15 and reference therein). For previous fire sites within the last decade, the thermal and hydraulic parameters for slope soil are parameterized using an empirical relationship^66,67^ that considers organic matter in the soils. The USGS National Land Cover Dataset 2001 (NLCD 2001, Ref. 68) is used for setting up the slope upper layer’s biomass loading and root system interferences for slope hydrology and soil mechanics. Depending on the degree of burning, the litter from surface vegetation also is parameterized according to a biomass-allocating inventory (see Ref. 69; of ~90 m resolution) and the approach of Ref. 70. The MODIS vegetation indices and NPP products are also used in setting up lower limits of soil thickness profiles on the slopes. Forests, shrubs and bare ground areas are specified with a 1, 0.5 and 0.05 m thickness, respectively. This approach is complementary to the scheme proposed by Detrich et al. (1995)^25^ and used in setting up the initial soil depth profile. Another complement to the original SHALSTAB scheme is that both the DEM and its Hessian (2^nd^-order derivative) are now considered in SEGMENT-Landslide. The soil depths at various known locations fit well with the direct measurements.

The unified treatment of SEGMENT-Landslide on flow is performed in the following interlinked steps.

1. Apply the DEM, establish the initial sliding material depth, initial moisture content, and soil and vegetation parameters
2. With time-varying precipitation, integrate the momentum equations (with parameterized mechanical properties of the sliding material) to obtain the flow fields
3. Diagnose the entrainment process involved and adjust the sliding material depth and update the DEM (step 1). Thus, a feedback loop forms. Soil moisture profiles and the associated mechanical properties also are adjusted.
4. Processes cease when velocity fields become zero in the entire simulation domain (e.g., all sliding material, whether newly generated or pre-exist are depleted; and the new DEM configuration is now stable with evolved conditions; and sliding material deposits on the plateaus and kinetic energy is completely exhausted as heat).

For applications to the region of interest, initialization of soil moisture is simplified by the preceding fire and is set as the permanent wilting point. There is a general lack of soil depth surveys. In setting up the initial soil depth profile, the DEM and its Hessian (2^nd^ derivative) are considered, based on SHALSTAB. The location of the groundwater water table is set relative to the surface elevation of Lake Casitas.

**Table S1.** Characteristics of forty-three GHCN-D v2 quality rainfall stations around Montecito

| Station ID | Latitude/Longitude (˚N/˚E) | Elev./Dist. to “*M”*  (m)/(km) | Station ID | Latitude/Longitude (˚N/˚E) | Elev./Dist. to “*M”*  (m)/(km) |
| --- | --- | --- | --- | --- | --- |
| ***USC00047888*** | 33.74;-117.87 | 41/179.1 | ***USC00046719*** | 34.15;-118.14 | 263/139.4 |
| ***USC00047953*** | 34.0;-118.5 | 4.3/113.8 | ***USC00045064*** | 34.65;-120.45 | 23/79.5 |
|  | 01/01/1981-09/30/2010 | |  |  |  |
| USC00049325 | 34.54;-117.31 | 871/212.2 | USC00047470 | 33.95;-117.39 | 256/212.3 |
| USC00048839 | 35.02;-118.75 | 434.3/102.2 | USC00047473 | 33.97;-117.36 | 300/214.2 |
|  |  |  |  | 01/01/1981-09/30/2009 | |
| ***USC00047902*** | 34.42;-119.68 | 1.5/6.7 | ***USC00047785*** | 34.1;-118.1 | 137.2/144.5 |
|  |  |  |  | 01/01/1981-09/30/2010 | |
| ***USC00047851*** | 35.31;-120.66 | 94/134.7 | ***USC00046175*** | 33.60;-117.88 | 3/185.5 |
| ***USC00049152*** | 34.07;-118.44 | 131.1/115.9 | USC00042941 | 34.71;-118.4 | 932.7/112 |
| ***USC00046399*** | 34.45;-119.23 | 227.1/36 | ***USC00046943*** | 35.16;-120.7 | 11.9/125.3 |
| USC00046624 | 34.59;-118.1 | 796.1/140.6 | ***USC00046154*** | 34.95;-119.68 | 658.4/55.9 |
| USC00049452 | 35.59;-119.35 | 89/129.4 | ***USC00044647*** | 33.54;-117.78 | 13.4/196.6 |
|  |  |  |  | 01/01/1981-08/31/2010 | |
| ***USC00045866*** | 35.37;-120.84 | 36/151.5 | USC00044671 | 34.25;-117.19 | 1586/223 |
|  |  |  |  | 01/01/1981-08/31/2010 | |
| ***USC00041194*** | 34.19;-118.35 | 199.6/120.2 | ***USC00046006*** | 34.23;-118.07 | 1740.4/144 |
| USC00041244 | 35.40;-119.47 | 68.6/107.3 | ***USC00042214*** | 34.01;-118.41 | 28/121 |
| ***USC00041253*** | 34.58;-119.98 | 242.3/36.4 | ***USC00046730*** | 35.63;-120.69 | 222.5/163.3 |
| USC00044278 | 35.65;-117.82 | 741/211 | USC00042771 | 34.59;-117.63 | 899.2/182 |
|  |  |  |  | 01/01/1981-10/31/2010 | |
| ***USC00049785*** | 34.18;-118.57 | 240.8/100.3 | USC00047253 | 35.37;-117.65 | 1088/206.6 |
| USW00003159 | 34.74;-118.21 | 712.6/132.9 | ***USW00023190*** | 34.43;-119.84 | 2.7/20.5 |
| ***USW00023129*** | 33.81;-118.15 | 9.4/152.7 | ***USW00093111*** | 34.12;-119.12 | 4/58.9 |
| ***USW00023273*** | 34.9;-120.45 | 73.8/91 | ***USW00093134*** | 34.05;-118.24 | 70/134.5 |
| ***USW00023174*** | 33.94;-118.39 | 29.6/126.4 | ***USW00093209*** | 35.67;-120.63 | 247/164.1 |
| USW00023187 | 34.74;-118.72 | 1374.6/88.4 | ***USW00003122*** | 33.8;-118.34 | 27/137.6 |
| USW00023155 | 35.43;-119.05 | 149/121.4 |  |  |  |

**Table S2.** List of 24 CMIP5 models used in this study

| Model name (abbreviations in the context) | Model group/center | Horizontal Resolution (global grids) |
| --- | --- | --- |
| ACCESS1.0 | CSIRO-BoM | 192×145 |
| ACCESS1.3 | CSIRO-BoM | 192×145 |
| CCSM4 | NCAR | 288×192 |
| CESM1-BGC | NSF-DoE-NCAR | 288×192 |
| CESM1-CAM5 | NSF-DoE-NCAR | 288×192 |
| CNRM-CM5 | CNRM-CERFACS | 256×128 |
| FGOALS-g2 | LASG-IAP | 128×60 |
| GFDL-CM3 | NOAA/GFDL | 144×90 |
| GFDL-ESM2G | NOAA/GFDL | 144×90 |
| GFDL-ESM2M | NOAA/GFDL | 144×90 |
| GISS-E2-R | NASA/GISS | 144×90 |
| GISS-E2-H | NASA/GISS | 144×90 |
| HadGEM2-AO | MOHC | 192×145 |
| HadGEM2-CC | MOHC | 192×145 |
| HadGEM2-ES | MOHC | 192×145 |
| INM-CM4 | INM | 180×120 |
| IPSL-CM5A-LR | IPSL | 96×96 |
| IPSL-CM5A-MR | IPSL | 144×143 |
| IPSL-CM5B-LR | IPSL | 96×96 |
| MIROC-ESM | U. Tokyo, NIES and JAMSTEC | 128×64 |
| MIROC5 | U. Tokyo, NIES and JAMSTEC | 256×128 |
| MRI-CGCM3 | MRI | 320×160 |
| NorESM1-M | NCC | 144×96 |
| NorESM1-ME | NCC | 144×96 |

**Table S3.** Sixty debris-prone locations and the collecting basin characteristics for a 4-in in 3-hr precipitation scenario

| **Lat.** | **Lon.** | **Area** | **%Capac.** | **Ave.slope** |  | **Lat.** | **Lon.** | **Area** | **% Capac.** | **Ave.slope** |
| --- | --- | --- | --- | --- | --- | --- | --- | --- | --- | --- |
| 33.301 | -117.311 | 1.04 | 75 | 15.87 |  | 34.662 | -118.477 | 0.87 | 20 | 18.04 |
| 33.495 | -117.311 | 1.04 | 80 | 15.87 |  | ***34.856*** | ***-120.033*** | ***0.72*** | ***50*** | ***19.45*** |
| ***33.689*** | ***-117.505*** | ***1.25*** | ***100*** | ***27.20*** |  | 34.856 | -119.838 | 1.44 | 80 | 21.66 |
| 33.689 | -117.311 | 0.84 | 50 | 18.51 |  | 34.856 | -119.255 | 0.69 | 30 | 14.52 |
| 33.884 | -117.699 | 0.88 | 30 | 12.78 |  | ***34.856*** | ***-119.061*** | ***1.26*** | ***75*** | ***19.49*** |
| 34.078 | -118.866 | 1.19 | 20 | 11.51 |  | 34.856 | -118.866 | 0.96 | 70 | 21.23 |
| 34.078 | -118.672 | 0.75 | 80 | 24.77 |  | 35.051 | -120.422 | 1.04 | 37 | 17.75 |
| 34.078 | -118.477 | 0.81 | 20 | 10.64 |  | 35.051 | -120.227 | 1.07 | 20 | 13.50 |
| 34.273 | -118.283 | 1.10 | 80 | 17.77 |  | 35.051 | -119.449 | 0.87 | 24 | 13.93 |
| 34.273 | -118.088 | 1.32 | 90 | 24.90 |  | 35.051 | -118.672 | 1.04 | 20 | 9.45 |
| 34.273 | -117.699 | 1.27 | 95 | 25.90 |  | ***35.245*** | ***-120.811*** | ***1.07*** | ***80*** | ***22.10*** |
| ***34.273*** | ***-117.505*** | ***0.99*** | ***39*** | ***23.00*** |  | 35.245 | -120.616 | 1.25 | 24 | 8.05 |
| 34.273 | -117.311 | 1.18 | 100 | 27.02 |  | 35.245 | -120.422 | 0.98 | 98 | 25.08 |
| 34.467 | -120.422 | 0.90 | 20 | 11.69 |  | 35.245 | -120.227 | 0.85 | 25 | 10.65 |
| 34.467 | -120.033 | 0.80 | 20 | 10.45 |  | ***35.245*** | ***-118.477*** | ***1.16*** | ***60*** | ***21.56*** |
| 34.467 | -119.838 | 1.10 | 20 | 9.52 |  | 35.245 | -118.283 | 0.87 | 40 | 16.36 |
| 34.467 | -119.644 | 0.96 | 95 | 26.10 |  | 35.439 | -120.811 | 1.14 | 20 | 12.77 |
| ***34.467*** | ***-119.449*** | ***0.74*** | ***73*** | ***26.50*** |  | 35.439 | -120.422 | 0.76 | 20 | 13.49 |
| 34.467 | -119.255 | 0.84 | 39 | 15.64 |  | 35.439 | -119.838 | 0.90 | 20 | 12.59 |
| 34.467 | -119.061 | 0.70 | 40 | 18.21 |  | 35.439 | -118.672 | 1.16 | 20 | 13.87 |
| 34.467 | -118.866 | 1.15 | 46 | 19.94 |  | 35.439 | -120.422 | 0.76 | 20 | 13.49 |
| 34.467 | -118.283 | 0.84 | 20 | 12.78 |  | 35.439 | -119.838 | 0.90 | 20 | 12.59 |
| 34.467 | -118.088 | 0.96 | 20 | 10.01 |  | 35.439 | -118.672 | 1.16 | 20 | 13.87 |
| 34.662 | -120.227 | 0.77 | 20 | 13.30 |  | 35.439 | -117.505 | 0.92 | 40 | 15.06 |
| 34.662 | -120.033 | 0.72 | 20 | 12.48 |  | ***35.634*** | ***-120.811*** | ***0.84*** | ***30*** | ***17.66*** |
| 34.662 | -119.838 | 1.39 | 95 | 22.53 |  | 35.634 | -120.616 | 0.97 | 20 | 12.60 |
| ***34.662*** | ***-119.644*** | ***0.99*** | ***60*** | ***18.57*** |  | 35.634 | -120.422 | 1.16 | 30 | 13.69 |
| 34.662 | -119.449 | 1.24 | 20 | 15.06 |  | 35.634 | -118.672 | 1.51 | 100 | 21.30 |
| 34.662 | -119.255 | 1.05 | 50 | 14.82 |  | 35.634 | -118.477 | 1.35 | 40 | 18.77 |
| 34.662 | -118.866 | 0.85 | 20 | 17.92 |  | 35.051 | -119.838 | 0.80 | 20 | 14.73 |


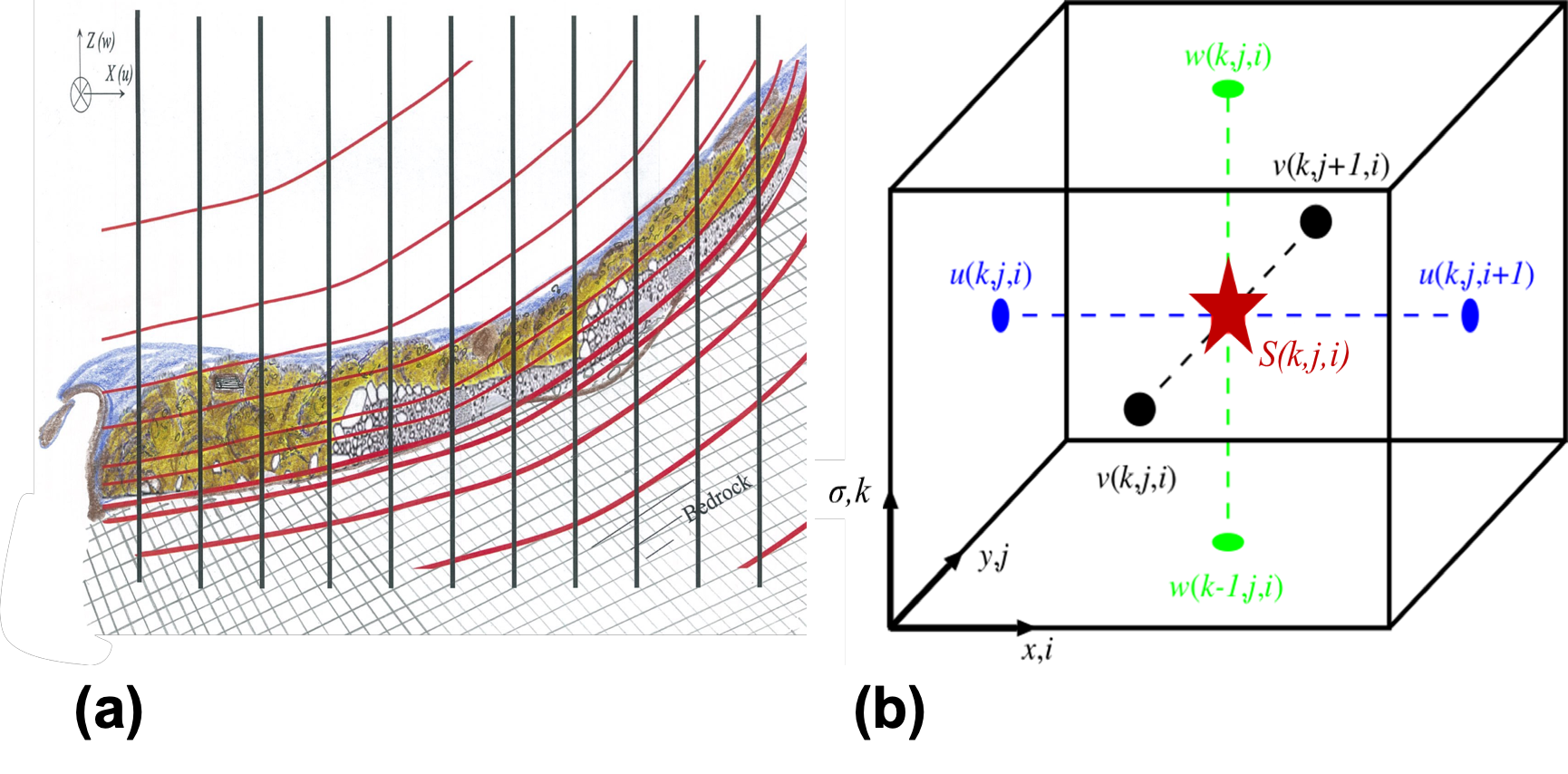


Figure S1: Grid stencils of the SEGMENT-Landslide modelling system. Red lines in (a) are stretched vertical levels. Default settings include 51 layers representing a vertical domain of a 135 m thick mantle of depth comprising 15 m below ground and 120 m above ground. Potential sliding materials are well confined in this simulation domain for the entire simulation timespan. In this cross-sectional view, the vertical lines are actually the lat/lon grid mesh. Staggering of mass and velocity fields is achieved through the Arakawa C grid stencil (Panel (b)), where scalars (*S*) and vertical velocities (*w*) are surrounded by horizontal flow vectors (u and v). The coordinate *x* points to local east, *y* points to local north, and *σ* is pointing vertically upward. The *i*, *j*, and *k* are the respective integer counts in the three dimensions.


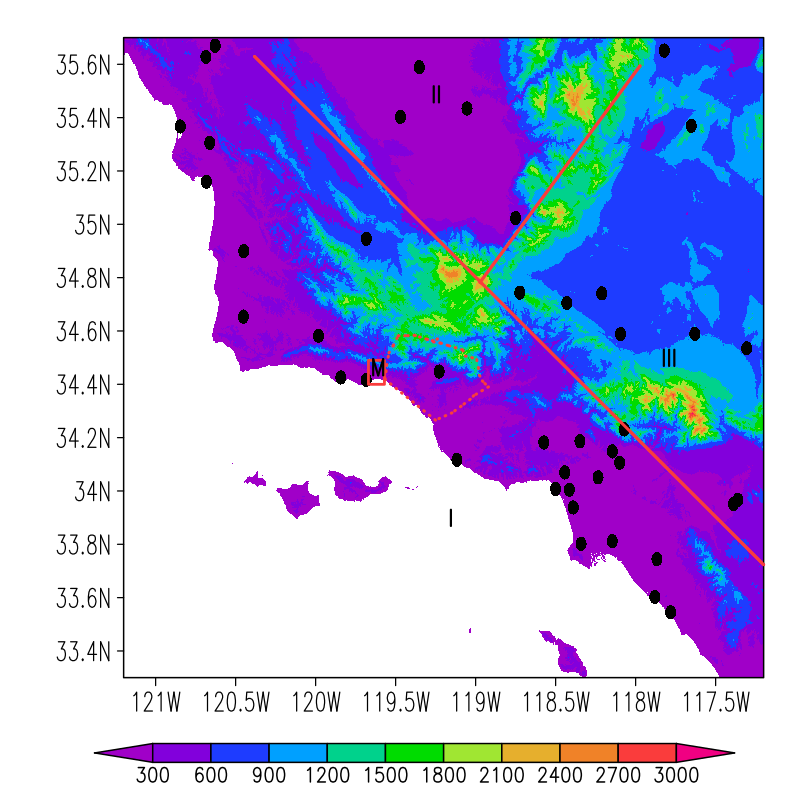


Figure S2: GHCN-D v2 weather stations (black dots) in the vicinity of our region of interest (red-box highlighted area; labelled with “M” for Montecito). The color shades of the background map are surface topography (m). The closest station is USC00047902, which is ~6.7 km southwest to the center of our region of interest (“M”). There are 43 weather stations in this 2.4º × 4º latitude/longitude region. Strong orographic effects make the rainfall highly non-uniform in this region. The red lines divide the region into three zones. Weather stations distributed within each zone are distributed more or less uniformly and are thus considered representative. Their averages will be used to compare with climate model simulations to investigate changes in rainfall morphology over the region of interest. The red dotted lines define the severely burned region (i.e., with complete above-ground biomass removal).


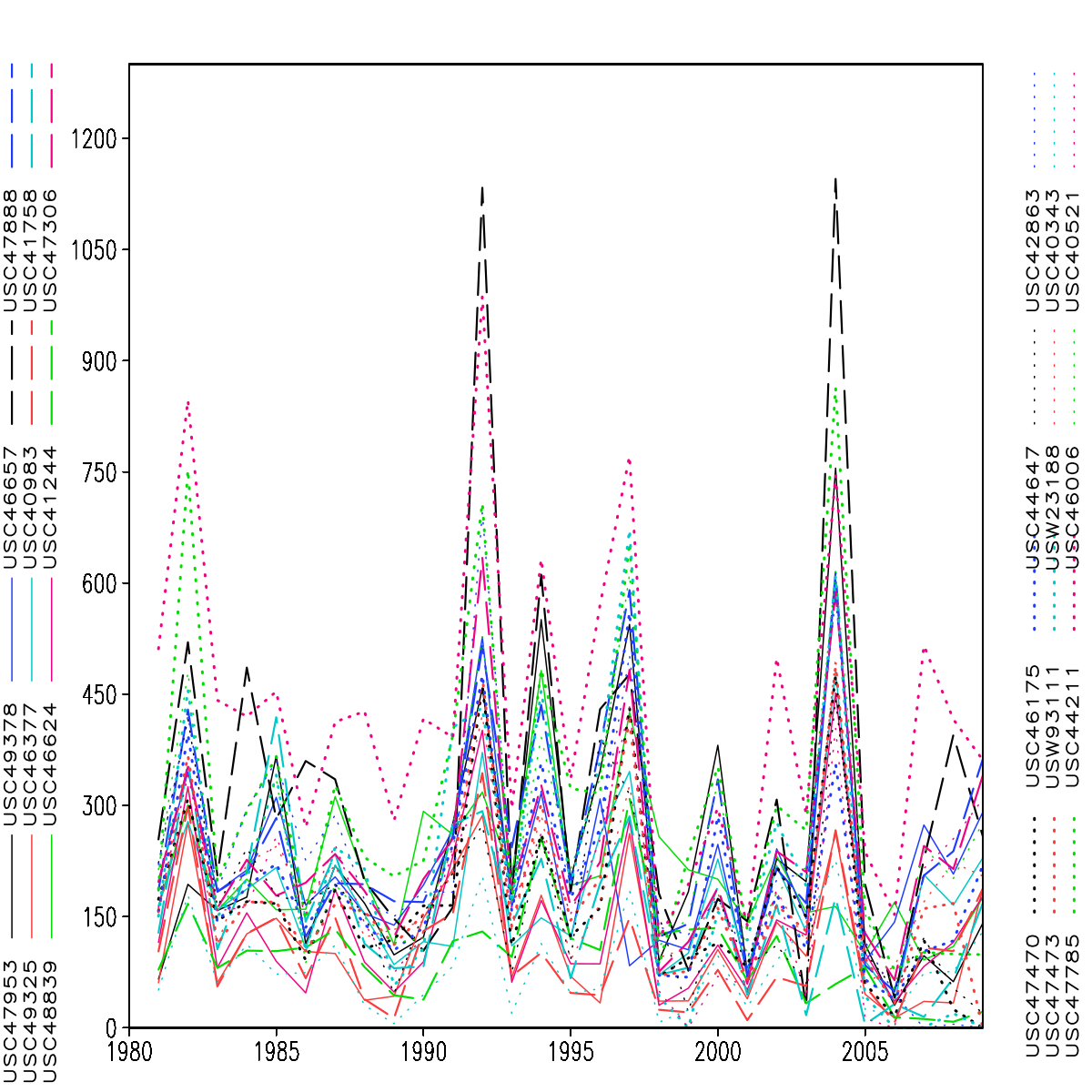


(a)


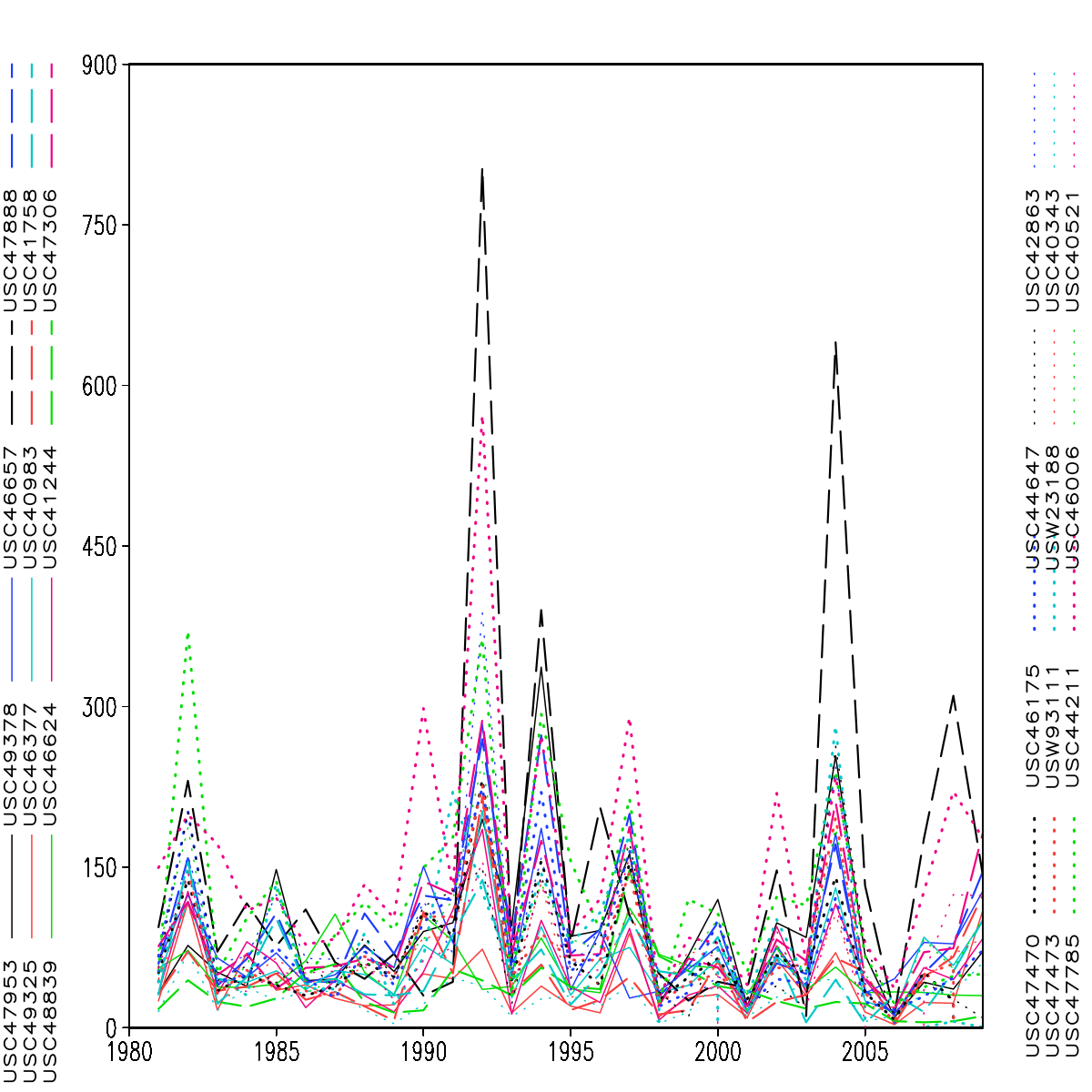


(b)

Figure S3: Total rainy season (October 01 to the following March 01 next year) rainfall totals (mm) over 24 selected stations around the region of interest (a), and the strongest rain event in the rainy season (b). Stations are listed in Table S1 for their location, elevation and observational period for the daily precipitation.

| (a) |
| --- |
| **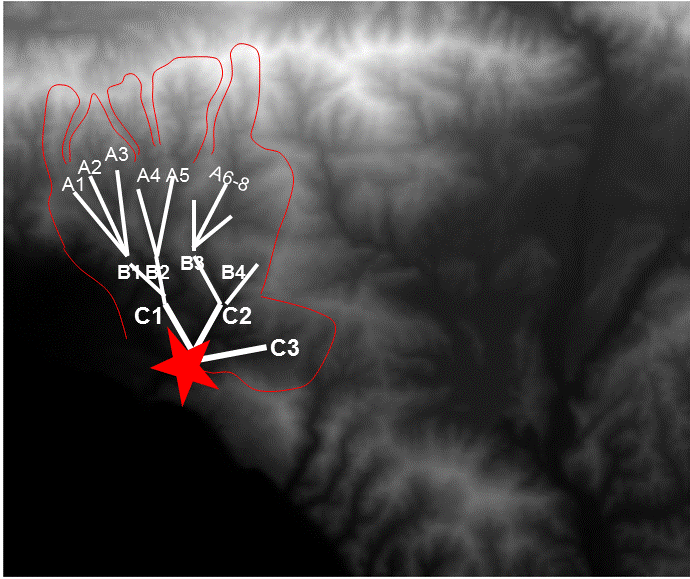**  (b) |
|  |

Figure S4: Conceptualized sketches of the Montecito debris flow of January 2018. Panel (a) illustrates the graded slope feature: steeper at higher elevations and becoming gentler at the toe. Against Carpenteria topography, panel (b) conceptualizes the universal feature of collecting basins prone to debris-flows. It is a 15km by 15 km regional area centered at 34.4ºN 119.45ºW, illustrating collection basins of the “progressive bulking” type for storm-triggered landslides. Clearly discernible from the 10-m resolution DEMs, the slope of Montecito is a south-facing graded slope. It also is an effective collecting basin in that small gullies merge into larger creeks of ever-increasing sizes. Within the basin size spectrum, the top three classes (i.e., width 600 m-2 km) are shown and denoted by: A (A1-A8), B (B1-B3) and C (C1, C2). Under significant runoff, the category ‘A’ gullies discharge into ‘B’ gullies and the latter further discharge into C’s, down the gravity potential. In this instance, gullies A1-A8 converge into lower elevation basins/creeks B1-B3, which further converge into even lower basins C1 and C2, forming mudslides at depositing region (star labelled). Many similar basin configurations occur along the coast.


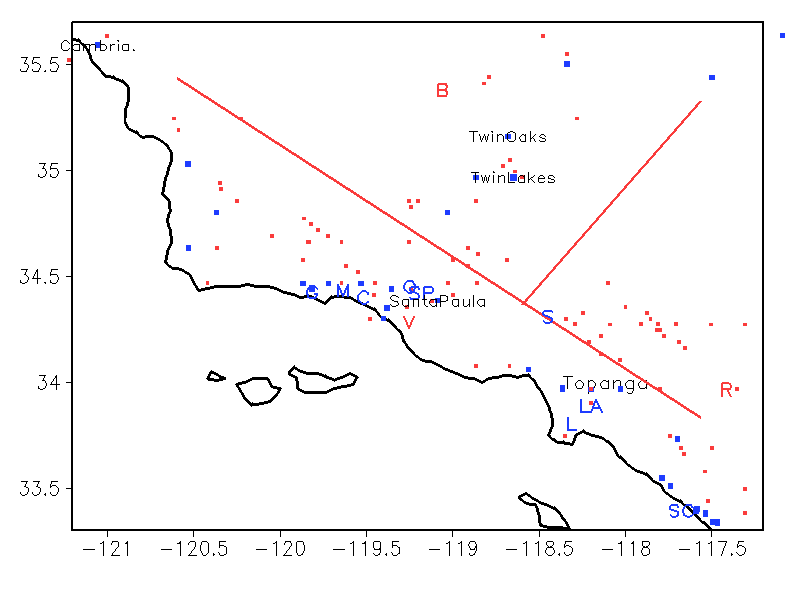


Figure S5: Large (involving more than 10^5^ m^3^ debris) debris flows simulated by SEGMENT-Landslide, with (all dots) and without (blue dots) boulder-rafting enhancement. Uniform precipitation 100 mm in a day is assumed. Boulder-rafting seems more decisive for the inland clusters of scarps (vegetation with higher biomass loads). Scarp size is exaggerated to make the locations legible. The red thick lines delineate three landslides clusters labelled with ‘B’, ‘V’ and ‘R’ as representative cluster centres. Major cities and town names are labelled using the abbreviations: ‘M’, ‘SP’, ‘O’, ‘S’, ‘C’, ‘R’, ‘B’, ‘V’, ‘SC’, ‘G’ and ‘LA’ stand respectively for Montecito, Steckel Park, Ojai, Sylmar; Carpinteria, Riverside, Bakersfield, Ventura, San Clemente, Goleta and Los Angeles.


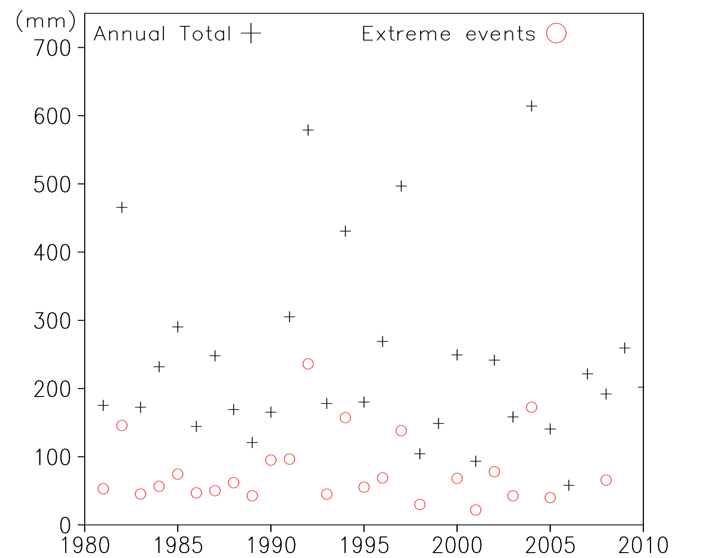


Figure S6: Total rainy season precipitation (crosses), from October 01 to March 01 (the following year), and the strongest rain event in the rainy season (circles). Stations are listed in Table S1 for their location, elevation and observation periods for daily precipitation. Precipitation over the 28 selected stations around the region of interest are weight-averaged using Cressman interpolation to obtain the rainfall over the region of interest.
